# An analysis of sexual dimorphism in the tumor microenvironment of colorectal cancer

**DOI:** 10.3389/fonc.2022.986103

**Published:** 2022-10-28

**Authors:** Andrea E. Geddes, Anita L. Ray, Robert A. Nofchissey, Azadeh Esmaeili, Apryl Saunders, Dawn E. Bender, Maaz Khan, Sheeja Aravindan, Jared T. Ahrendsen, Min Li, Kar-Ming Fung, Muralidharan Jayaraman, Jingxuan Yang, Kristina K. Booth, Gary D. Dunn, Steven N. Carter, Katherine T. Morris

**Affiliations:** ^1^ Department of Surgery, University of Oklahoma Health Science Center, Oklahoma City, OK, United States; ^2^ Department of Pathology, University of Oklahoma Health Science Center, Oklahoma City, OK, United States; ^3^ University of Oklahoma Health Science Center, Stephenson Cancer Center, Oklahoma City, OK, United States; ^4^ Department of Pathology, Beth Israel Deaconess Medical Center, Harvard Medical School, Boston, MA, United States; ^5^ Department of Medicine, University of Oklahoma Health Science Center, Oklahoma City, OK, United States; ^6^ Department of Cell Biology, University of Oklahoma Health Science Center, Oklahoma City, OK, United States

**Keywords:** colorectal cancer, tumor microenvironment, sexual dimorphism, T cell, immunoscore

## Abstract

Women with colorectal cancer (CRC) have survival advantages over men, yet the underlying mechanisms are unclear. T cell infiltration within the CRC tumor microenvironment (TME) correlates strongly with survival. We hypothesized that women with CRC have increased T cell infiltration and differential gene expression in the TME compared to men. Tissue microarrays comprising primary tumor, tumor infiltrated lymph nodes, and uninvolved colon were created from CRC patients. Proportions of CD4 positive (CD4+) and CD8 positive (CD8+) T cells were identified using immunohistochemistry. TME immune- and cancer-related genetic expression from primary and metastatic CRC tumor were also evaluated *via* the NanoStringIO360 panel and The Cancer Genome Atlas Project database. CD4+ was higher in tumor samples from women compared to men (22.04% vs. 10.26%, p=0.002) and also in lymph node samples (39.54% vs. 8.56%, p=0.001). CD8+ was increased in uninvolved colon from women compared to men (59.40% vs. 43.61%, p=0.015), and in stage I/II tumors compared to III/IV in all patients (37.01% vs. 23.91%, p=0.009). Top CD8+ tertile patients survived longer compared to the bottom (43.9 months vs. 25.3 months, p=0.007). Differential gene expression was observed in pathways related to Treg function, T cell activity, and T cell exhaustion, amongst several others, in women compared to men. Thus, significant sexual dimorphism exists in the TME that could contribute to survival advantages observed in female patients with CRC.

## Introduction

Colorectal cancer (CRC) is the third leading cause of cancer-related deaths worldwide and despite similar screening, medical therapy, and surgical management, a decreased incidence of disease and improved disease-related survival exists in women as compared to men (1–3). Hormonal, environmental, and immune-related differences have been investigated but the disparity in CRC outcomes between men and women is not completely understood (4–6). In all patients with CRC, however, improved disease-related survival has been linked to certain factors within the tumor microenvironment (TME).

The TME encompasses a complex, dynamic interaction between tumor cells and cells within the invasive margin. Tumor progression is not only dependent on the intrinsic tumor cell behavior but also on surrounding non-tumoral cell types and their behavior. Increased immune cell infiltration into the TME has been shown to provide defense against tumor progression and increased CD8 positive T cells in patients with CRC has been independently associated with improved disease-related survival (7). In fact, T cell infiltration, as measured by Immunoscore (combined density of CD8+ and CD3+ T cells in the tumor and invasive margins), has been validated as an even stronger assessment for prognosis of CRC cancer than the classic TNM staging system (7, 8). Similarly, the immune cell profile of the CRC TME may provide mechanistic insights and targetable treatment approaches using immunotherapy (9).

Given improved survival with increased TME T cell infiltration and longer survival in women as compared to men, our aim was to determine whether there were differences within the TME between women and men with CRC. We also sought to determine if a difference in immune cell activity exists in the TME by analyzing gene expression from primary and metastatic tumors from men and women with CRC. We hypothesized that women with CRC have increased T cell infiltration and differential expression of immune- and cancer-related genes within the TME as compared to men. To investigate this, we compared helper CD4 positive (CD4+) and cytotoxic CD8 positive (CD8+) T cell infiltration *via* immunohistochemistry in patient-derived primary tumors, lymph nodes involved by tumor metastasis, and uninvolved peri-tumoral colonic tissues between men and women. We then compared genetic expression differences in the TME by comparing primary and metastatic CRC tumor samples from men and women using a targeted immune-oncology gene panel. Finally, we compared gene expression profiles between men and women by examining data from The Cancer Genome Atlas Program (TCGA, Firehose Legacy) (10, 11).

## Materials and methods

### Patient cohorts and tumor specimens

Tissue from men and women with colon cancer who were treated at the University of Oklahoma Health Sciences Center and underwent oncologic resection between 2010-2015 were included for the immunohistochemical analysis *via* tissue microarray (TMA). Tissues from a different set of patients who underwent CRC resection at the University of Oklahoma Health Sciences Center from 2017-2019 were included for NanoString analysis to explore potential differences in genetic expression in the TME. Additional analysis of the publicly available TCGA Firehose Legacy dataset (10, 11) was incorporated to identify possible differential gene expression in CRC samples between men and women. Patient demographic data were retrieved by manual chart review, including date of surgery, age of patient at diagnosis, location of primary tumor, stage of disease, and status of mismatch repair genes expression by IHC (MLH1, MSH2, MSH6, and PMS2). Date of patient death or date of last contact was also recorded. Patients who died within one month of surgery were excluded from analysis.

Institutional review board (IRB) approval for waiver of consent was obtained for the TMA studies. Tumor samples used for gene expression profiling with NanoString were obtained after informed consent from the patients under a prospectively approved IRB.

### Immunohistochemistry

Tissue core samples from the tumor bed, involved lymph node, and uninvolved surrounding colon tissue were obtained from formalin fixed paraffin embedded tissue blocks. Tissue cores were arranged in a TMA, fixed in 10% formalin, and embedded in paraffin. The tissue blocks were then sliced into 5 µm thick sections with a microtome, transferred onto positively charged slides, dried overnight at room temperature, and incubated at 60°C for 45 minutes. This was followed by deparaffinization and rehydration in an automated multistainer (Leica ST5020). Sectioned tissue was processed and stained using antibodies against CD4 or CD8, according to manufacturer’s protocol, using a Leica Bond-IIITM Polymer Refine Detection system (DS 9800). Positive and negative controls were used according to standard institutional protocols.

Whole slides were digitally scanned and then analyzed using Aperio software to identify and quantify CD4+ and CD8+ T cells. The percent of cells staining for CD4 out of total cell counts is reported for CD4+ percent with the same approach for CD8+ cells. Two pathologists reviewed all tissue prior to digital analysis. Samples were excluded from analysis if greater than 80% tissue degradation was present, non-representative tissue was submitted (such as adipose tissue within the tumor block), or the tissue core was from a lymph node with less than 20% cancer involvement seen on microscopy.

### NanoString profiling and TCGA data

For NanoString profiling, tissue samples from primary and metastatic tumor were snap frozen at time of resection. Frozen tissues were homogenized and RNA was isolated and purified from tumor lysates using Direct-zol RNA miniPrep Plus per manufacturer protocol. RNA integrity was assessed to assure minimal degradation using the Agilent Bioanalyzer and samples were included only if there was at least 50% RNA over 200 BP in length. Genetic expression profiling of 770 genes was performed using the PanCancer IO360™ Panel from NanoString Technologies. Data was normalized to 15 housekeeping genes. Data analysis was performed *via* n-Solver Analysis software (IO 360 Data Analysis Service) per the NanoString platform standard protocols, which included positive and negative controls with internal housekeeping genes.

TCGA transcriptome data was accessed using cBioPortal for Cancer Genomics. Potential mean gene expression differences between men and women were assessed using TCGA Firehose Legacy RNA Seq V2 RSEM and mRNA microarray of CRC tumors (10, 11).

### Statistical analysis

Averages are reported as mean ± standard deviation. Means for continuous variables were compared with a two-way Student’s t-test. Frequency distributions for categorical data were compared with Fisher’s Exact test. Non-parametric survival analysis was performed using log-rank tests and Kaplan-Meier analysis. NanoString log2-transformed count data was analyzed *via* t-test. Statistical significance was defined as p-value less than 0.05.

## Results

Patients included in the TMA, NanoString, and TCGA analyses were well-matched between men and women, with no significant difference in the number of patients, patient age, location of tumor, or CRC stage with the exception of more samples from male patients in the TCGA data compared to those from female patients (**Table 1**). In the TMA population, 101 patients were included with an average age of 61.0 ± 13.1 years (range: 24-88 years). The NanoString population included 32 patients (after one was excluded due to low RNA integrity number), with an average age of 58.7 ± 12.6 years (range: 23-79 years), and the TCGA included 633 patients with average age of 66.0 ± 12.8 year (range: 31-90 years) (**Table 1**).

**Table 1:** Clinical characteristics of TMA, NanoString, and TCGA Firehose Legacy patient groups.

	All Patients	Women	Men	p-value
**Number**				
IHC	101	51	50	0.953
NanoString	32	17	15	0.501
Firehose	633	297	336	0.05
**Age (years)**				
IHC	60.9 ± 13.0	60.7 ± 14.0	61.1 ± 12.0	0.865
NanoString	58.7 ± 12.6	58.7 ± 12.6	58.8 ± 12.8	0.991
Firehose	66.0 ± 12.8	66.0 ± 13.8	66.0 ± 11.9	0.992
**Stage**				
IHC				0.513
I	23	12	11	
II	24	12	12	
III	43	21	22	
IV	11	6	5	
NanoString				0.253
I	5	4	1	
II	11	3	8	
III	4	3	1	
IV	10	6	4	
Unknown	2	1	1	
Firehose	109	48	61	0.104
I	230	109	121	
II	182	92	90	
III	91	40	51	
IV	21	8	13	
**MSI**				
IHC	5	3	2	0.902
NanoString	12.4 ± 9.5	10.6 ± 7.2	13.7 ± 10.9	0.504
**Survival (months)**				
IHC	41.0 ± 26.2	38.0 ± 23.8	45.0 ± 28.7	0.902
NanoString	12.4 ± 9.5	10.6 ± 7.2	13.7 ± 10.9	0.504

P-values represent comparison between women and men.

**Figure 1:**
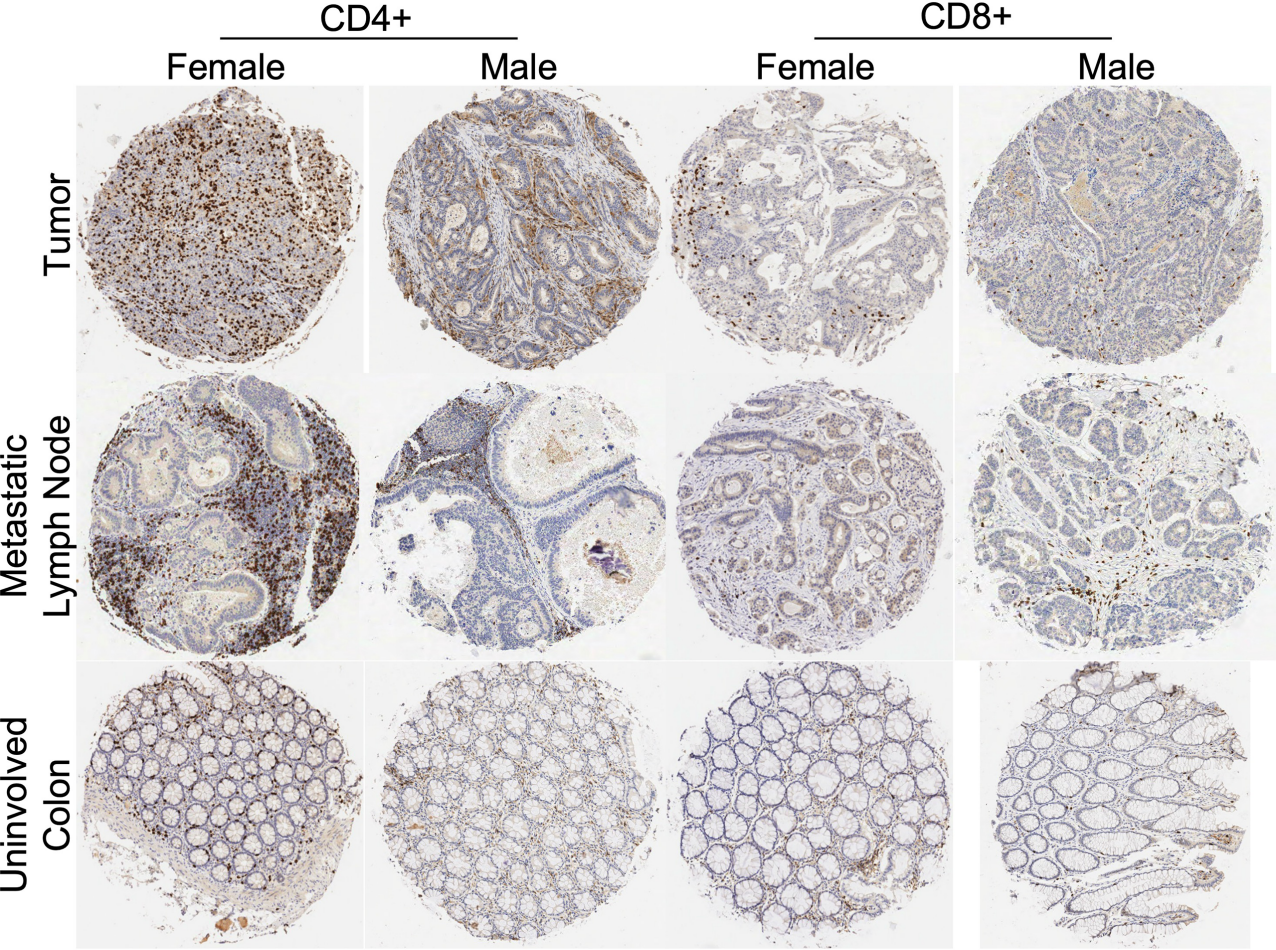
Tissue microarray histologic representations of colon tumor tissue, metastatic lymph node, and uninvolved colon from female and male patients after immunohistochemistry staining for CD4+ and CD8+ antibodies. Tissue from tumor specimens (top row), tissue from involved lymph node specimens (middle row) and tissue from uninvolved colon specimens (bottom row). Representative IHC from a female and male patient with stage III CRC.

**Figure 2:**
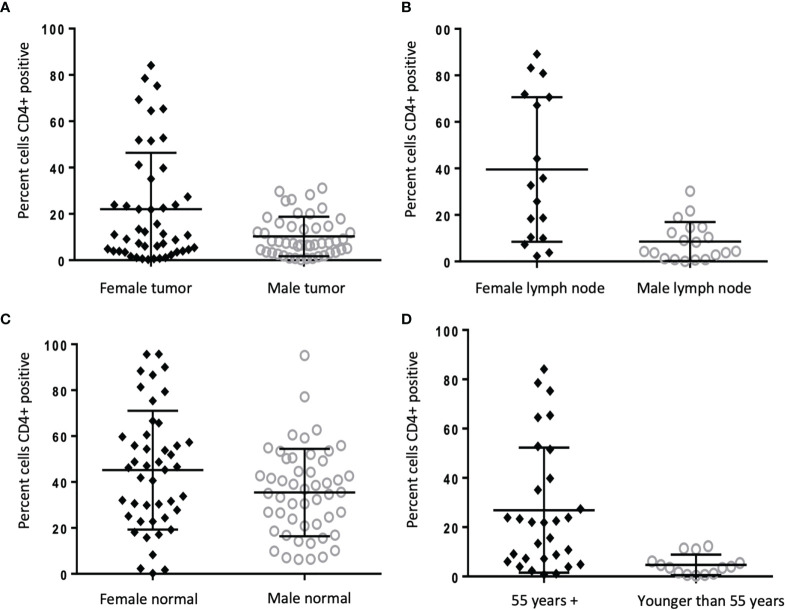
CD4+ cells increased in TME of women compared to men, and in older women compared to younger. **(A-C)** Frequency of CD4+ staining reported as percent cells positive in tumor (22.04% vs 10.26%, p=0.002), lymph node (39.54% vs 8.56%, p=0.001), and uninvolved colon tissue (3.9% vs 2.7%, p=0.045) from men (n = 50) and women (n = 51) with colon cancer. **(D)** Frequency of CD4+ staining reported as percent positive cells in tumor in women ages >55 (n = 30) compared to younger than 55 (n = 14) (26.9% vs. 4.7%, p=0.002).

Immunohistochemistry was performed using a TMA to identify percentage of CD4+ helper T cells and CD8+ cytotoxic T cells within the examined tissue (**Figure 1**). An increased percentage of CD4+ cells was seen in all tissues from women as compared to men, with more CD4+ in the tumor (22.04% vs. 10.26%, p=0.002), lymph nodes (39.54% vs 8.56%, p=0.001), and a modest but still significant increase of CD4+ cells in surrounding uninvolved colonic tissue (3.91% vs 2.72%, p=0.045) (**Figure 2A-C**). When women were stratified by age (≥55 years compared to <55 years), there was a significantly increased amount of CD4+ infiltration in tumor samples of older women compared to younger (26.90% vs. 4.71%, p=0.002) (**Figure 2D**); however, similar age differences were not observed in men.

**Figure 3:**
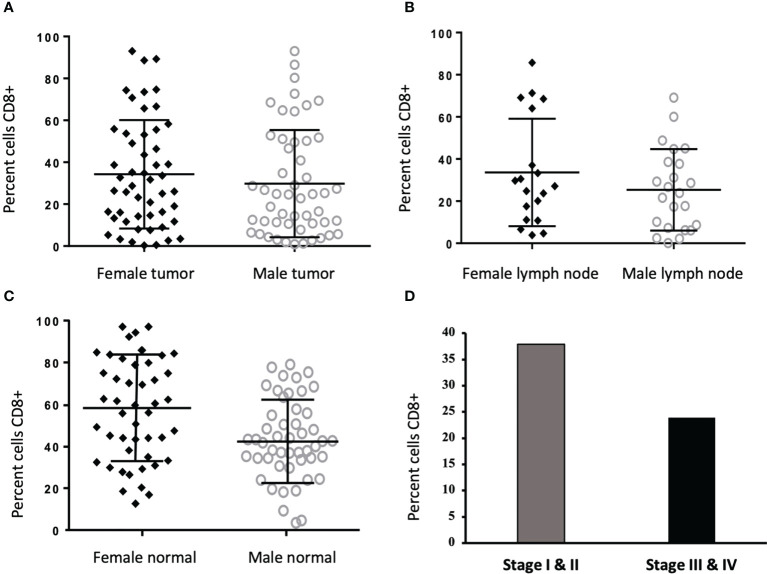
CD8+ cells are increased in normal colon tissue of women and in Stage I/II tumors. **(A-C)** Frequency of CD8+ staining reported as percent cells positive in tumor (34.77% vs. 29.81%, p=0.145) and lymph node (34.41% vs. 25.39%, p=0.591) were not different between men (n = 50) and women (n = 51), however, CD8+ was increased in normal tissue from women as compared to men (47.40% vs. 34.61%, p=0.015). **(D)** When stratified into cancer stages, CD8+ cells more frequently infiltrated tumors from stages I and II (n = 47) compared to III and IV (n = 54)(37.01% vs 23.91% p=0.009).

Between the sexes, there was no significant difference in CD8+ percentage in tumor (34.77% vs. 29.81%, p=0.145) or lymph tissue (34.41% vs. 25.39%, p=0.591);. Notably, CD8+ cells were increased in uninvolved colon of women as compared to men (59.40% vs. 43.61%, p=0.015) (**Figures 3A–C**), however, the effects of estrogen (E2) and estrogen receptor (ESR2) on CD8+ T cells cannot be ruled out. When stratified by tumor stage, higher numbers of CD8+ cells were found in stages I and II compared to III and IV (37.01% vs 23.91% p=0.009) (**Figure 3D**).

**Figure 4:**
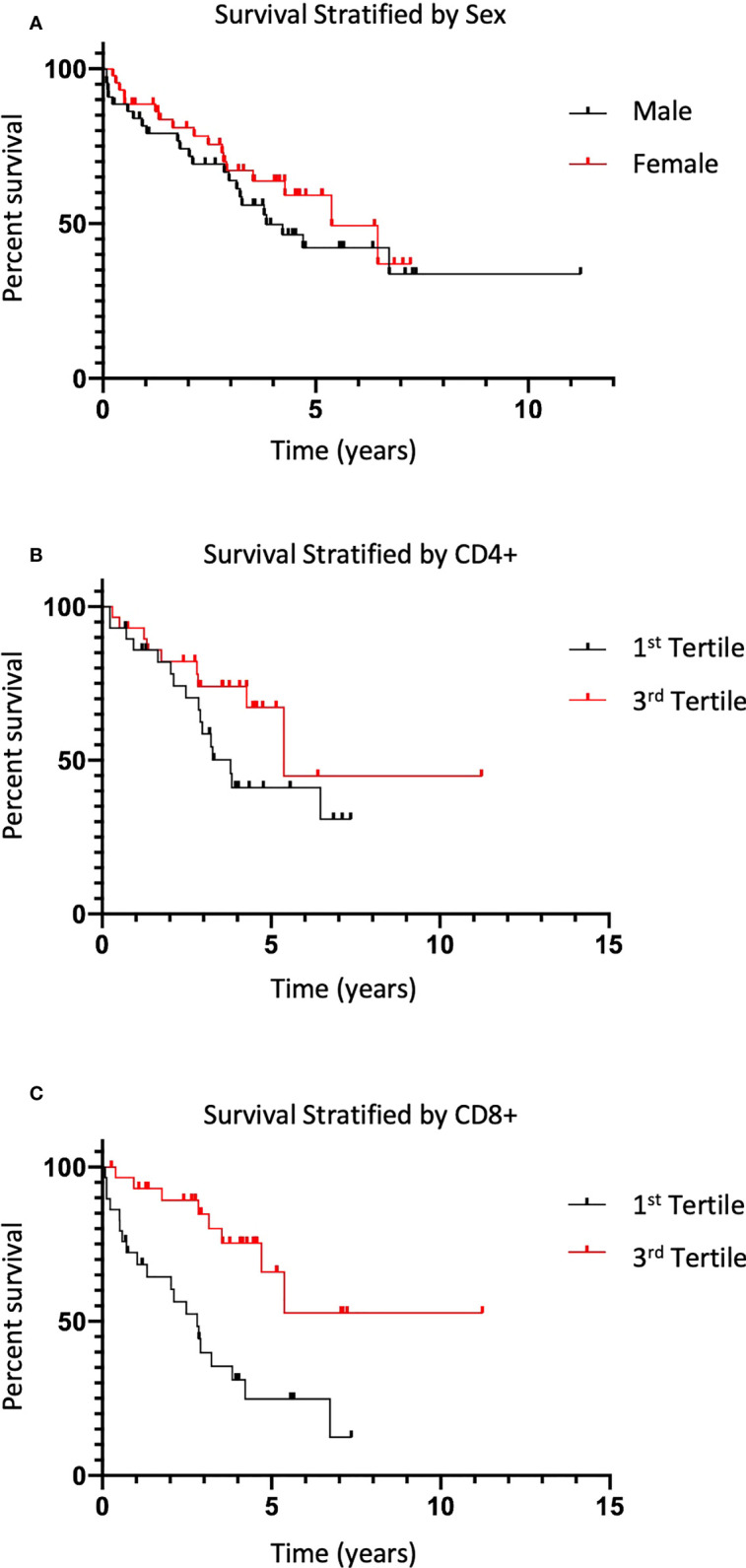
Survival is increased in patients with increased CD8+ infiltration. **(A)** Overall mean survival of CRC patients from the TMA dataset stratified by sex are shown (38 vs 45 months, p=0.361). **(B)** Average difference in survival as defined in **(A)** in patients within the 1^st^ tertile of CD4+ infiltration in tumor tissue compared to patients in 3^rd^ tertile (p=0.142). **(C)** Difference in survival as defined in **(A)** in patients with 1^st^ tertile CD8+ infiltration in tumor tissue compared to patients in 3^rd^ tertile (43.9 vs 25.3 months, p=0.007).

Kaplan-Meier analysis revealed no significant differences between patients within the first (high) tertile compared to the third (low) tertile of CD4+ infiltration in tumor tissue (**Figure 4B**). However, longer overall survival was observed in patients within the first (high) tertile versus third (low) tertile of CD8+ infiltration in tumor tissue (43.9 vs 25.3 months, p=0.007) (**Figure 4C**). This could be due to the co-association of higher CD8+ percentage with lower stage found in our study (**Figure 3D**). After analysis of overall survival of patients in the TMA and NanoString cohorts, there was no significant difference found between men and women (p=0.361) (**Figure 4A**).

**Figure 5:**
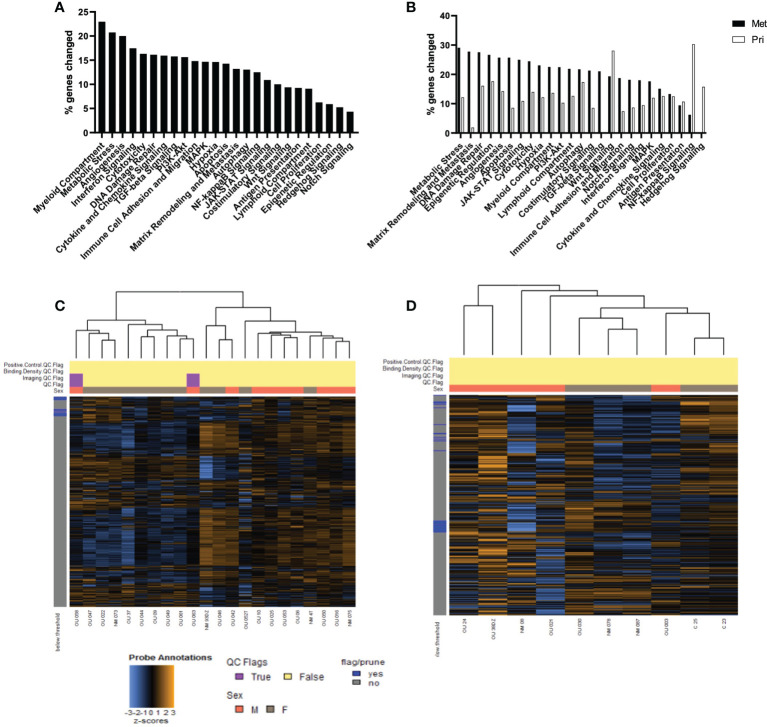
Gene expression differs in the TME of men and women in both primary and metastatic tumors. **(A)** The top percent of genes in pathways designated in annotated groups, as defined by NanoString technologies, which were significantly different in expression between women as compared to men (p<0.05). **(B)** Top percent of genes in pathways differentially expressed between men and women from primary tumor compared to metastatic tumor (p<0.05). Heatmap for NanoString analysis of primary **(C)** and metastatic **(D)** CRC samples. Gene expression levels are illustrated with a Z score. Orange indicates high expression; blue indicates low expression.

Using an independent cohort of tissue samples, we then performed genetic expression profiling of 770 genes in 25 broadly defined gene sets using the PanCancer IO360™ Panel from NanoString Technologies, similar to a prior study (12). The analysis revealed multiple differentially expressed genes in all gene sets. Using gene expression from men as the referent population, the gene sets with the greatest number of genes differentially expressed in women were related to the myeloid compartment (23.01%), metabolic stress (20.70%), angiogenesis (20.02%), interferon signaling (17.53%), cytotoxicity (16.31%), DNA damage repair (16.14%), and cytokine and chemokine signaling (16.01%) (p<0.05 for each) (**Figure 5A**). Furthermore, sex-associated differences in genetic expression were exaggerated in almost every pathway examined in metastatic tissue samples compared to primary tumor tissue (p<0.05) (**Figure 5B**). Summary heatmaps for the primary and metastatic samples are shown in **Figures 5C, D**, respectively. Additionally, mismatch repair deficiency (MMR) was assessed in this cohort, revealing three patients (two men, one woman) with MMR deficient tumors (data not shown).

Within the differentially expressed genes of metastatic tumors, there were clusters of genes related to the regulation of T regulatory (Treg) cells, IFN-γ response, and CD8+ exhaustion. Of the Treg-related genes, tissue from women showed increased expression of *GOT1* (10.11-fold, p=0.004) and *GHR* (7.61-fold, p=0.001) and decreased expression of *DAB2* (0.38-fold, p=0.013), *TNFRSF25* (0.49-fold, p=0.045), and *LRRC32* (0.29-fold, p=0.019) compared to men (data not shown).

Alongside these observed decreases in Treg-related genes, there were increases in genes related to IFN-γ production, Th1 activity, and cytotoxic CD8+ activity in tissue from women. Specifically, Th1-associated *IL18R1* (6.13-fold, p=0.012), *GBP1* (3.50-fold, p=0.021), and *STAT4* (2.80-fold, p=0.021) showed significantly increased expression in tumors from women compared to men. Co-stimulatory markers of T cells including *CD96* (2.80-fold, p=0.021), *DPP4* (4.01-fold, p=0.001), *GZMK* (4.21-fold, p=0.042), and *CCL14* (4.71-fold, p=0.005) were also elevated in tumors from women. Interestingly, the shifts in Treg and anti-tumor T cell expression were accompanied by increased expression of the exhaustion markers *PD-L1* (3.83-fold, p=0.011), *TIGIT* (3.61-fold, p=0.011), and tumor suppressor *FBP1* (9.23-fold, p=0.013) in women.

**Figure 6:**
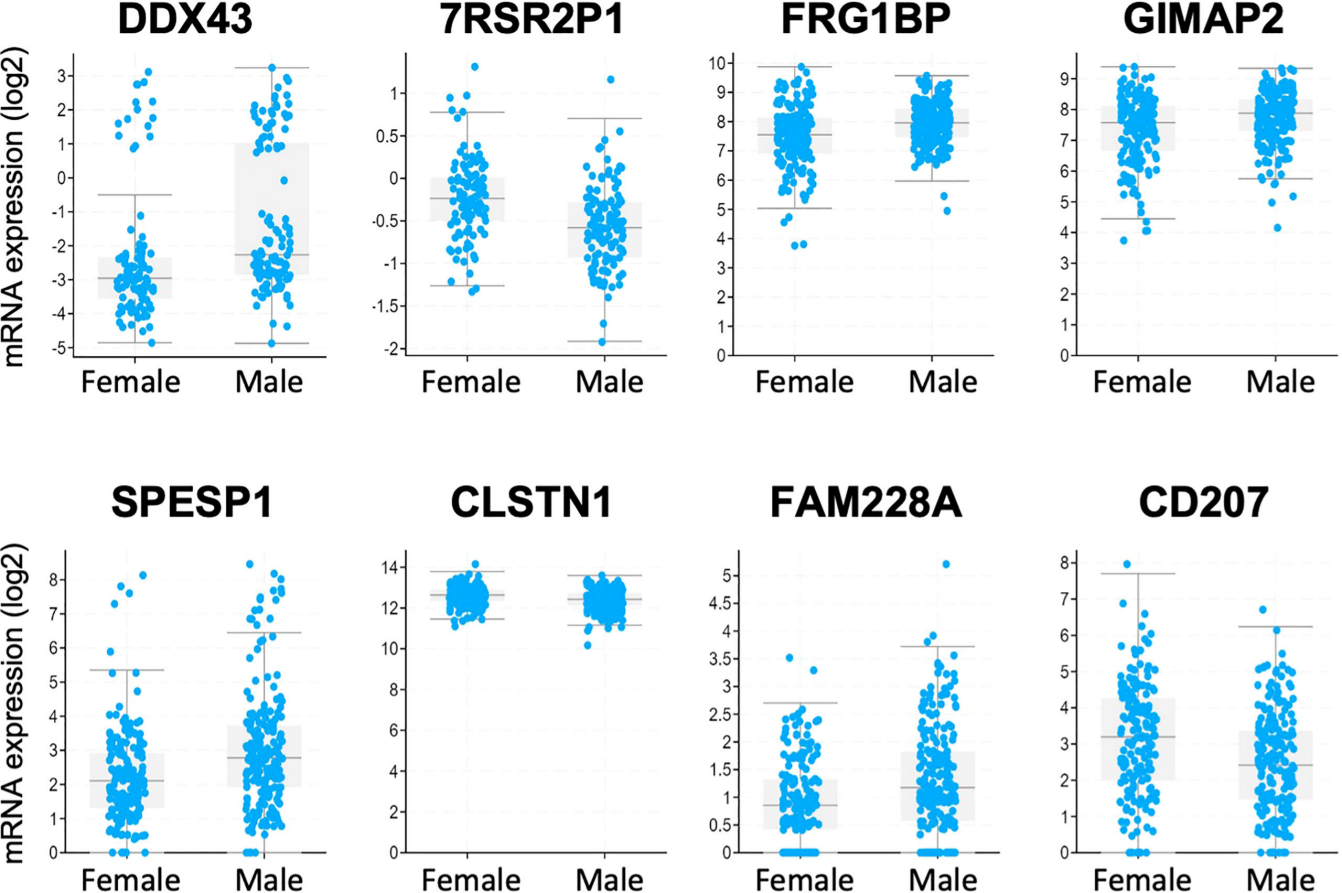
Analysis of the TCGA Firehose Legacy transcriptome data of CRC patient samples identified genes that were differentially expressed between men and women. Interestingly, only 8 of the 65 identified genes were located outside the sex chromosomes.

Analysis of the Firehose Legacy dataset in TCGA revealed a total of 65 genes which demonstrated significant differential expression between men (n=335) and women (n=294) with CRC, with 57 of these genes being present on the X or Y chromosomes. **Figure 6** demonstrates the differential expression of the 8 genes not found on X or Y chromosomes: *DDX43*, *ZRSR2P1*, *FRG1BP*, *GIMAP2*, *SPESP1*, *CLSTN1*, *FAM228A*, and *CD207*.

## Discussion

Colon cancer remains a leading cause of cancer related death in both men and women; however, women experience longer disease-related survival compared to men. In an equally stage-distributed population, the mortality rate for men is almost 50% higher for men (10.8 per 100,000 person-year) than for women (7.2 per 100,000 person-year) (13). The mechanisms underlying this sex-based difference in survival have yet to be fully elucidated. Multiple studies support the role of female sex hormones reducing the development and progression of colon cancer in women. A study from the Surveillance Epidemiology and End Results (SEER) found that women under 45-years of age (presumably pre-menopausal) with metastatic CRC lived longer than age-matched men with CRC (14). In addition, a decreased incidence of CRC was seen in postmenopausal women taking combined estrogen and progesterone compared to those not on hormone therapy (14, 15). Similarly, the mechanistic role of E2 and ESR2 in providing survival advantage in women with CRC has been demonstrated (16–18). However, it is unclear what role female sex hormones have in men with regards to tumor development, and the inconsistency of hormonal influences on incidence and survival in men with CRC suggests mechanisms of survival beyond hormonal effects on the tumors (19). On the other hand, T cell infiltration within the TME is a validated prognostic factor independently associated with increased overall survival in all populations (7). We therefore sought to determine if there was a different immune response in the TME between men and women by direct counting of CD4+ and CD8+ T cells, and by profiling immune and cancer related gene expression between sexes.

We show here that women with CRC have increased CD4+ T cells in tumor tissue, uninvolved peri-tumoral tissue, and metastatic lymph nodes as compared to men. Another study found increased CD4+ memory activated T cells in T1-2 tumors compared to T3-4, as well as in tumor involved tissue compared to uninvolved tissue (9); however, this study did not assess for sex-related TME differences in CRC. Activated memory CD4+ T cells have been shown to promote maturation and activation of other immune cells such as B cells, macrophages, and cytotoxic CD8+ cells (20). In another study, the total CD4+ T cell count was reduced within TME of invasive margins compared to uninvolved tissue (21). Interestingly, higher CD4+ infiltration was associated with higher tumor stage and lymph node spread and also improved overall survival in patients with stage I-III non-small cell lung cancer (NSCLC) (22).Together with our results, these findings could indicate that increased CD4+ T-lymphocytes may be associated with tumors of lower T stage, decreased invasiveness, and less aggressive disease, perhaps by means of E2 and ERbeta on CD4+ T cells in the TME (16, 17). However, the sexual dimorphism of the TME is likely more complex, with influences from multiple factors, including circulating hormones, genetics, nutrition, environment, metabolism, and intrinsic tumor properties.

We also explored the effects of hormonal influences by stratifying women by age (55 years) because 95% of women greater than 55 years old have undergone menopause (23, 24). Interestingly, women >55 years had increased CD4+ in tumor tissue compared to younger women, suggesting that lower estrogen and progesterone levels may be associated with increased CD4+ T cell infiltration into the TME, similar to what has been observed in NSCLC (25). This age-based difference, although preliminary, provides possible evidence of hormonal contributions to CD4+ helper T cell infiltration beyond just the sex-related differences we observed. Similar age-related differences were not seen in tissue samples from men.

CD8+ T cell infiltration was also increased in uninvolved peri-tumoral tissue of women as compared to men and has been previously suggested to represent an independent prognostic factor in stage II/III CRC (26). Infiltrating cytotoxic CD8+ T cells exhibit numerous anti-tumoral functions, and our findings of increased infiltration into tissues surrounding the tumor suggests increased immune involvement and therefore better defense against tumor burden and metastasis in women compared to men (27). This is supported by prior findings of CD8+ cell infiltration into the TME as a predictor of improved clinical outcome by Immunoscore methodology (7). Increased CD8+ T cells in uninvolved tissue from women could represent a potential mechanism underlying the decreased incidence of CRC among women as well as the survival advantage observed in women CRC patients.

After analysis of survival, although increased infiltration of CD4+ was seen in all tissue compartments in samples from women, there was no clear relationship between CD4+ infiltration and overall survival in this study. Specifically, we did not find a survival difference in patients between the top tertile and bottom tertile of CD4+ infiltration. This highlights the complexity underlying the TME, with multiple factors contributing to the sexual dimorphism seen in CRC. Although CD4+ T cells may play an important role in the TME, the amount of CD4+ T cell infiltration may not be a clear prognostic indicator. On the other hand, increased CD8+ T cell infiltration was found in tumors from patients with stages I/II disease as compared to III/IV, and increased CD8+ was also associated with increased survival in both men and women in our study. The increased survival we saw in patients with increased CD8+ is likely due to the co-association of higher CD8+ counts with lower stage. In our overall cohort, survival between men and women at all stages and within each stage was not significantly different. However, a limitation of this study is the relatively small sample size, which is likely insufficient to allow for a robust analysis of survival. Similarly, these results indicate likely sex-independent mechanisms of CD8-dependent CRC survival in men and women.

Differential gene expression was seen in our analysis of a second cohort of patients using the PanCancer IO360™ Panel from NanoString Technologies, as well as from data gathered from TCGA Firehose Legacy Dataset. Interestingly, many genes with differential expression between men and women from the TCGA data were located on sex chromosomes. The NanoString data revealed a larger number of differentially regulated immune-related genes within metastatic tumors taken from women and men as compared to primary tumors. This is of particular interest given that metastatic disease is a major contributor to mortality in CRC. Thus, these data highlight a potential link between the immune response and metastatic tumor behavior, which could contribute to improved overall survival in women with CRC [reviewed previously (28)].

Within the differentially expressed genes of metastatic tumors, we found clusters of genes related to the management of regulatory T (Treg) cells, IFN-γ response, and CD8+ T cell exhaustion. For example, *GARP* expression was significantly decreased in women in our study. *GARP* expression has been shown to modulate the function of Treg cells in the colon such that mice with Treg-specific *GARP* deletion developed fewer colon tumors and less tumor burden compared to wild-type mice (29). Thus, differences in Treg-related gene expression could contributed to sexual dimorphism in the TME of CRC (30).

We also show that expression of *CD96*, a co-stimulatory marker in T cells, was elevated in metastatic tumors from women. Increased *CD96* has been associated with better overall survival in patients with CRC and has been suggested as a potential biomarker for prognosis in CRC (31). Furthermore, we show a higher expression of *CCL14* in tumors from women. Suppression of *CCL14* has been shown previously to contribute to CRC progression *via* the Wnt/B-catenin pathway (32). These findings suggest that increased T cell activity is related to better immune defense against tumor cells in the TME of women compared to men, similar to that of advanced lung cancer (33).

Together, the changes we observed across multiple cancer pathways and T cell responses in particular suggests that there are multiple sex-associated differences in the TME, as we have shown previously using a murine model of metastatic CRC (34). These findings of differential gene expression in metastatic tissue emphasizes the importance of increasing the number of samples from metastatic disease within publicly available sequencing datasets, so that differences between primary and metastatic tumors can be more fully examined.

Although we found increased CD4+ and CD8+ T cell density in tissue from women, a limitation of this study is that we did not evaluate activity or phenotype of T cells within the TME. A possibility exists that these cells are immunosuppressive or may even be in a functionally exhausted state (35). However, we did find differential genetic expression patterns that support the conclusion that there is sexual dimorphism in the TME of CRC, with differences in genetic expression that support a potentially more advantageous local immune response in women.

Analysis of T cell infiltration and immune-related gene transcription within the TME of CRC reveals a clear sexual dimorphism within the immune response to CRC that warrants additional investigation and is in keeping with previously described sexual dimorphism in CRC (3, 36). If the mechanisms behind this dimorphic response can be identified, new therapeutic immunologic targets might be found. Future directions include identifying the phenotype of the CD4+ and CD8+ T cells within the TME *via* immunoprofiling and examining specific pathways responsible for increased T cell infiltration within tumors, lymph nodes, and peritumoral histologically normal mucosa from women with CRC.

## Data availability statement

Datasets are available on request: The raw data supporting the conclusions of this article will be made available by the authors, without undue reservation.

## Ethics statement

The studies involving human participants were reviewed and approved by Institutional Review Board (IRB) approval for waiver of consent was obtained for the TMA studies. Tumor samples used for gene expression profiling with NanoString were obtained after informed consent from the patients under a prospectively approved IRB. The patients/participants provided their written informed consent to participate in this study.

## Author contributions

Study design: AG, AR, AS, KM. Data collection and analysis: AG, AR, AS, RN, AE, DB, MK, SA, JA, ML, KB, GD, SC, KM. Manuscript writing, critical review, editing: AG, AR, AS, RN, AE, DB, MK, SA, JA, MJ, KB, GD, SC, KM. All authors contributed to the article and approved the submitted version.

## Funding

Research reported in this publication was supported in part by the National Cancer Institute Cancer Center Support Grant P30CA225520 awarded to the University of Oklahoma Stephenson Cancer Center and used the Laboratory of Molecular Biology and Cytometry Research and the Biospecimen and Tissue Pathology Shared Resources. In addition, the work was also supported by the University of Oklahoma Health Sciences Department of Surgery. The content is solely the responsibility of the authors and does not necessarily represent the official views of the National Institutes of Health.

## Acknowledgments

Immunohistochemistry and TMA construction services provided by the Cancer Tissue Pathology Shared Resources was supported partly by the National Institute of General Medical Sciences Grant P20GM103639 and National Cancer Institute Grant P30CA225520 of the National Institutes of Health.

## Conflict of interest

The authors declare that the research was conducted in the absence of any commercial or financial relationships that could be construed as a potential conflict of interest.

## Publisher’s note

All claims expressed in this article are solely those of the authors and do not necessarily represent those of their affiliated organizations, or those of the publisher, the editors and the reviewers. Any product that may be evaluated in this article, or claim that may be made by its manufacturer, is not guaranteed or endorsed by the publisher.
